# EEG Signal Classification Using Manifold Learning and Matrix-Variate Gaussian Model

**DOI:** 10.1155/2021/6668859

**Published:** 2021-03-25

**Authors:** Lei Zhu, Qifeng Hu, Junting Yang, Jianhai Zhang, Ping Xu, Nanjiao Ying

**Affiliations:** School of Automation, Hangzhou Dianzi University, Hangzhou 310000, China

## Abstract

In brain-computer interface (BCI), feature extraction is the key to the accuracy of recognition. There is important local structural information in the EEG signals, which is effective for classification; and this locality of EEG features not only exists in the spatial channel position but also exists in the frequency domain. In order to retain sufficient spatial structure and frequency information, we use one-versus-rest filter bank common spatial patterns (OVR-FBCSP) to preprocess the data and extract preliminary features. On this basis, we conduct research and discussion on feature extraction methods. One-dimensional feature extraction methods like linear discriminant analysis (LDA) may destroy this kind of structural information. Traditional manifold learning methods or two-dimensional feature extraction methods cannot extract both types of information at the same time. We introduced the bilinear structure and matrix-variate Gaussian model into two-dimensional discriminant locality preserving projection (2DDLPP) algorithm and decompose EEG signals into spatial and spectral parts. Afterwards, the most discriminative features were selected through a weight calculation method. We tested the method on BCI competition data sets 2a, data sets IIIa, and data sets collected by our laboratory, and the results were expressed in terms of recognition accuracy. The cross-validation results were 75.69%, 70.46%, and 54.49%, respectively. The average recognition accuracy of new method is improved by 7.14%, 7.38%, 4.86%, and 3.8% compared to those of LDA, two-dimensional linear discriminant analysis (2DLDA), discriminant locality property projections (DLPP), and 2DDLPP, respectively. Therefore, we consider that the proposed method is effective for EEG classification.

## 1. Introduction

Brain-computer interface (BCI) is a kind of real-time communication system connecting the brain and external devices. BCIs based on electroencephalogram (EEG) can convert the information sent by the brain into commands to drive the external devices, so as to realize the communication between people and the outside world [1]. There are several control signal types in BCI and, among them, motor imagery (MI) is one of the most studied applications [2]. MI is an independent BCI method that uses motor cortex as a signal source. The user imagines moving his/her limbs without any actual muscular movement. Studies on EEG signal indicate that when people perform motor imaging tasks, this will cause an event-related desynchronization (ERD) and event-related synchronization (ERS) of oscillations in alpha band (8–13 Hz) and beta band (14–30 Hz) [3]. Due to these characteristics, researchers can process and analyze EEG signals in relevant frequency bands for the classification of motor imaging tasks.

In MI-based BCI system, a well-known problem is how to handle very large amounts of features extracted from multichannel EEG signals. Feature extraction is a commonly used approach for solving this problem. To solve the computational complexity and data storage problem caused by the high dimension of signals, many dimensionality reduction methods have been used in traditional BCI technology. Kumar et al. [4] used independent components analysis (ICA) to remove artifacts in EEG signals and used principal component analysis (PCA) for reducing high-dimensional data. Although PCA seeks to learn a projection that can preserve the main energy of data, it does not contain discriminability. Linear discriminant analysis (LDA) uses the label information to enlarge the between-class distance and reduce the within-class distance [5]. Although LDA uses the label information in MI, it ignores the important local information in the EEG signal. Motor imaging EEG signals are collected through electrodes spread over the cerebral cortex. When a certain imaging task is performed, ERD/ERS will occur in specific local areas of the brain [6]. There are mutual influences and connections between the signals collected by adjacent electrodes. These connections exist not only in the spatial position but also in the frequency domain. As described above, the main focus is on alpha band (8–13 Hz) and beta band (14–30 Hz). The LDA method cannot effectively extract these local features. Since the manifold learning method was proposed [7, 8], some studies assumed that EEG signals are more likely to exist in low-dimensional nonlinear manifold subspace. Since then, manifold learning methods have been applied on epileptic EEG signals [9, 10], EEG-based depth of anesthesia assessment [11], emotional state classification [12], tracking dynamic EEG brain connectivity [13], and so forth. At the same time, manifold learning was applied in MI-based BCI system [14–16].

The goal of methods based on manifold learning is to keep locality-geometric structure of data in a neighboring area and successfully find the inherent features existing in nonlinear manifold [17]. Locality property projections (LPP) [18], neighborhood preserving embedding (NPE) [19], and locally linear embedding (LLE) [8] are the popular methods of manifold learning, which preserve the locality property. But they suffer from a limitation that they deemphasize discriminant information which is important in recognition problem. Discriminant locality property projection (DLPP) [20] aims to find the subspace that best discriminates different classes by minimizing the within-class distance, while maximizing the between-class distance. However, they are all vector-based methods. EEG signals, as multichannel data, contain rich spatial information. The spatial information here means that the EEG signal is in the form of a matrix, and the matrix contains two-dimensional information closely related to the spatial structure. Significant spatial channel information will be lost by using vector-based methods [21]. According to these factors, the two-dimensional dimensionality reduction method is considered. Two-dimensional discriminant locality preserving projection (2DDLPP) [22] is the 2D expansion of DLPP. 2DDLPP can extract two-dimensional information and preserve geometric structures of original data. But, for EEG signals, both spatial information and spectral information are important information that is effective for classification. 2DDLPP only calculates a single-sided projection and cannot use the spatial and spectral characteristics of the EEG signal at the same time.

In addition to the manifold learning method, sparse learning has been increasingly applied to feature extraction. For example, sparse linear discriminant analysis (SLDA) [23] and sparse two-dimensional discriminant locality preserving projection (S2DDLPP) [17] are proposed to learn a sparse discriminant subspace for feature extraction. Researches have shown that the method based on sparse can effectively reduce the computational complexity and has good robustness to noise [24–26]. But the above methods are somewhat sensitive to the selection of the number of dimensions, since the discriminability of each projection direction is fixed [27].

To solve the above issues and combine the important local information in the EEG signal with the 2D matrix processing method, we propose an extension of 2DDLPP based on the Gaussian variable model. The main idea of matrix-variable Gaussian model [28] implies a separable structure for the covariance matrix of the vectorized data and it shows that the covariance between any two spatial-spectral features can be decomposed into two terms. On this basis, we calculate the eigenvalues and eigenvectors of spatial term and spectral term, respectively. After that, the two sets of eigenvalues are multiplied in pairs and sorted, and those features with a large weight are selected. This allows the spectral and spatial characteristics to be analyzed at the same time, thereby ensuring that the extracted features have the best discriminativeness.

This paper proposes a bilinear two-dimensional discriminant locality preserving projection (B2DDLPP) algorithm that based on a matrix-variable Gaussian model. Compared with 2DDLPP, B2DDLPP uses a bilinear structure to fully extract the connections between the channels of the EEG signal. Both spatial information and frequency information are considered at the same time, so it is more suitable for the task of feature extraction of motor imaging EEG signals.

## 2. Materials and Methods

### 2.1. Related Methods

The input data of the feature extraction algorithm introduced in this paper is the feature matrix after OVR-FBCSP, and each sample gets a feature matrix with a size of *N*_*f*_ × *N*_*g*_, where *N*_*f*_ is the number of frequency bands in FBCSP (9 in this article), *N*_*g*_= 2 × *m* × Numclass, *m* is the number of pairs of features, and Numclass is the total number of classes. When using the vector-based feature extraction method, we vectorize the feature matrix of each sample as input. The class of each feature matrix is the same as the corresponding sample; and the OVR-FBCSP algorithm will be introduced in Section 2.3.1.

#### 2.1.1. Discriminant Locality Property Projections (DLPP)

DLPP is based on the extension of LPP, and it considers the discrimination information. This makes DLPP have better performance on classification problems compared to LPP.

The objective function of DLPP is as follows:

(1) $$\begin{array}{c} \frac{\sum_{s=1}^{Z}\sum_{i,j=1}^{n_{s}}{\left(y_{i}^{s}-y_{j}^{s}\right)}^{2}W_{ij}^{s}}{\sum_{i,j=1}^{Z}{\left(m_{i}-m_{j}\right)}^{2}B_{ij}}, \end{array}$$

where *Z* is the number of motor imagination classes, *n*_*s*_ is the number of samples in the *s*th class, **y**_*i*_^*s*^ denotes the *i*th weight vector in the *s*th class, **m**_*i*_ and **m**_*j*_ are separately the mean weight vectors for the *i*th class and *j*th class, respectively; that is, **m**_*i*_=(1/*n*_*i*_)∑_*k*=1_^*n*_*i*_^**y**_*k*_^*i*^ and **m**_*j*_=(1/*n*_*j*_)∑_*k*=1_^*n*_*j*_^**y**_*k*_^*j*^, where *n*_*i*_ and *n*_*j*_ are the numbers of samples in the *i*th class and *j*th class, separately. **W**_*ij*_^*s*^ is the weight between **y**_*i*_^*s*^ and **y**_*j*_^*s*^, and **B**_*ij*_ is the weight between **m**_*i*_ and **m**_*j*_. It should be noted that *s* in **y**_*i*_^*s*^ and **W**_*ij*_^*s*^ is the upper corner mark, not power operation.

Suppose that **a** is a transformation vector; that is, *Y* = aTX. By simple algebra formulation, the objective function can be turned to

(2) $$\begin{array}{c} \frac{a^{T}XLX^{T}a}{a^{T}FHF^{T}a}, \end{array}$$

where *L* = D-W, and W can be defined as **W**_*ij*_^*s*^=exp(−‖**x**_*i*_^*s*^ − **x**_*j*_^*s*^‖^2^/*t*). *D* is a diagonal matrix, and its entries are column (or row) sum of Ws; **D**_*ii*_^*s*^=∑_*j*_**W**_*ij*_^*s*^. *H* = E-B, and B can be defined as **B**_*ij*_^*s*^=exp(−‖**f**_*i*_ − **f**_*j*_‖^2^/*t*), where **f**_*i*_ is the mean value of samples in the *i*th class; *F* = *f*1, *f*2,…,fs; *E* is a diagonal matrix, and its entries are column (or row) sum of B; **E**_*ii*_=∑_*j*_**B**_*ij*_.

DLPP subspace is spanned by a set of vectors **a**, satisfying

(3) $$\begin{array}{c} a=\arg\min\frac{a^{T}XLX^{T}a}{a^{T}FHF^{T}a}. \end{array}$$

The numerator of objective function reflects within-class distance, while the denominator reflects between-class distance. The vectors **a**_*i*_ that minimize the objective function are given by minimum eigenvalues solutions to the generalized eigenvalues problem.

Thus, *A* = [**a**_1_, **a**_2_,…, **a**_*d*_] are the solutions of equation (3), ordered according to their eigenvalues, *λ*_0_, *λ*_1_,…, *λ*_*d*−1_; and the embedding is as follows:

(4) $$\begin{array}{c} x_{i}⟶y_{i}=A^{T}x_{i}. \end{array}$$

#### 2.1.2. Two-Dimensional Discriminant Locality Preserving Projection (2DDLPP)

2DDLPP is the 2D expansion of DLPP. The main advantage of 2DDLPP over DLPP is that it has a more accurate approximation of the original signals, which can avoid the losses of important information for recognition. The objective function of 2DDLPP is

(5) $$\begin{array}{c} J\left(Y\right)=\frac{\sum_{s=1}^{Z}{\sum_{i,j=1}^{n_{s}}\left(Y_{i}^{s}-Y_{j}^{s}\right)}^{T}\left(Y_{i}^{s}-Y_{j}^{s}\right)W_{ij}^{s}}{\sum_{i,j=1}^{Z}{\left(M_{i}-M_{j}\right)}^{T}\left(M_{i}-M_{j}\right)B_{ij}}, \end{array}$$

where **Y**_*i*_^*s*^ and **Y**_*j*_^*s*^ denote the projected feature matrices in class *s*, corresponding to the original EEG signals. *Z* is the number of motor imagination classes. **W**_*ij*_^*s*^ and **B**_*ij*_ are the within-class weight matrix and the between-class weight matrix, separately. **M**_*i*_ and **M**_*j*_ represent the mean matrices of the projected signals in class *i* and class *j*; that is, **M**_*i*_=(1/*n*_*i*_)∑_*k*=1_^*n*_*i*_^**Y**_*k*_^*i*^ and **M**_*j*_=(1/*n*_*j*_)∑_*k*=1_^*n*_*j*_^**Y**_*k*_^*j*^.

Suppose that *X* is an *N*_*f*_ × *N*_*g*_ feature matrix signal and A denotes the transformation matrix. The linear transformation is *Y* = ATX.

The objective function can be reformed as follows:

(6) $$\begin{array}{c} J\left(A\right)=\frac{A^{T}XLX^{T}A}{A^{T}FHF^{T}A}=\frac{A^{T}P_{w}A}{A^{T}P_{b}A}, \end{array}$$

where

(7) $$\begin{array}{c} P_{w}=\frac{1}{2}\sum_{s=1}^{Z}\sum_{i,j=1}^{n_{s}}\left(X_{i}^{s}-X_{j}^{s}\right){\left(X_{i}^{s}-X_{j}^{s}\right)}^{T}W_{ij}^{s},\\ P_{b}=\frac{1}{2}\sum_{i,j=1}^{Z}\left(F_{i}-F_{j}\right){\left(F_{i}-F_{j}\right)}^{T}B_{ij}, \end{array}$$

where **F**_*i*_ is the mean matrix of the *i*th class; it is defined as **F**_*i*_=(1/*n*_*i*_)∑_*k*=1_^*n*_*i*_^**X**_*k*_^*i*^. B is the weight matrix between any two classes' mean matrix and it is defined as **B**_*ij*_=exp(−‖**F**_*i*_ − **F**_*j*_‖^2^/*t*). **W**_*s*_ is the weight matrix between any two samples in the *s*th class, and it is defined as

(8) $$\begin{array}{c} W_{ij}^{s}=\exp\left(\frac{-{\left\|X_{i}^{s}-X_{j}^{s}\right\|}^{2}}{t}\right), \end{array}$$

where *t* is a parameter that can be set empirically and in this paper it is set to 1.

*L* = *D* − W is the Laplacian matrix, where Ds is a diagonal matrix, and its entries are column (or row) sum of Ws; **D**_*ii*_=∑_*j*_**W**_*ji*_; and *H* = *E* − *B*, where *E* is a diagonal matrix, and its entries are column (or row) sum of B; **E**_*ii*_=∑_*j*_**B**_*ji*_.

The projection directions can be obtained by minimizing the objective function, satisfying

(9) $$\begin{array}{c} A=\arg\underset{A}{\min}\frac{A^{T}XLX^{T}A}{A^{T}FHF^{T}A}. \end{array}$$

The minimization problem can be turned into a generalized eigenvalue problem:

(10) $$\begin{array}{c} XLX^{T}A=\lambda FHF^{T}A. \end{array}$$

Thus, *A* = *a*1, *a*2,…, ad are the solutions of (9), ordered according to their eigenvalues, *λ*_0_, *λ*_1_,…, *λ*_*d*−1_.

### 2.2. Matrix-Variate Gaussian Model and Its Combination with Manifold Learning

#### 2.2.1. Matrix-Variate Gaussian Model

The matrix-variate Gaussian model is the normal distribution of the matrix. Let *f*(**X***|*Ω_*i*_) denote the conditional probability of matrix X∈*ℝ*^*N*_*f*_×*N*_*g*_^ under class Ω_*i*_, and the matrix-variate Gaussian model for matrix **X** is denoted by

(11) $$\begin{array}{c} X|\Omega_{i}∼ℳN_{N_{f}\times N_{g}}\left(M_{i},\phi_{i},\psi_{i}\right),\;1\leq i\leq Z, \end{array}$$

where *Z* is the total number of classes, the matrices *M*_*i*_ denote the mean matrix of the classΩ_*i*_, and *ϕ*i and Ψi denote covariance matrix of the class Ω_*i*_. In this paper, *ϕ*i and Ψi denote spectral covariance and spatial covariance, respectively. These matrices are defined as follows:

(12) $$\begin{array}{c} M_{i}=E_{X|\Omega_{i}}\left(X\right),\\ \phi_{i}={\text{tr}}^{-1}\left(\psi_{i}\right)*E_{X|\Omega_{i}}\left(\left(X-M_{i}\right){\left(X-M_{i}\right)}^{T}\right),\\ \psi_{i}={\text{tr}}^{-1}\left(\phi_{i}\right)*E_{X|\Omega_{i}}\left({\left(X-M_{i}\right)}^{T}\left(X-M_{i}\right)\right). \end{array}$$

The conditional probability of matrix *X* can be determined by Mi, *ϕ*i, and Ψi as follows:

(13) $$\begin{array}{c} f\left(X|\Omega_{i}\right)=\frac{\exp\left\{-\left(1/2\right)\text{tr}\left[\phi_{i}^{-1}{\left(X-M_{i}\right)}^{T}\psi_{i}^{-1}\left(X-M_{i}\right)\right]\right\}}{{\left(2\pi \right)}^{\left(N_{f}N_{g}\right)/2}\det{\left(\phi_{i}\right)}^{\left(N_{f}/2\right)}\det{\left(\psi_{i}\right)}^{\left(N_{g}/2\right)}}, \end{array}$$

where det(·) represents the determinant of a matrix.

We define column vectorization as vec (·); then the mean of matrix *X* equals vec (Mi). By vectorizing the matrix Gaussian distribution, it can be converted into a multivariate Gaussian distribution of vector data as follows:

(14) $$\begin{array}{c} X∼ℳN_{N_{f}\times N_{g}}\left(M,\phi ,\psi \right)\Leftrightarrow \text{vec}\left(X\right)∼N\left(\text{vec}\left(M\right),\psi ⊗\phi \right), \end{array}$$

where *ϕ*∈*ℝ*^*N*_*f*_×*N*_*f*_^, Ψ∈*ℝ*^*N*_*g*_×*N*_*g*_^, and ⊗ represents the Kronecker product operator. It can be seen from equation (14) that the covariance matrix of vectorized data can be transformed into a separable structure, which consists of the Kronecker product of two matrices.

The matrix-variate model in equation (11) corresponds to a specific structure for the covariance of the vectorized data. This model implies that the covariance matrix of the vectorized data can be decomposed into two parts. This separability property will be used in the algorithms proposed in this paper.

#### 2.2.2. Bilinear Two-Dimensional Discriminant Locality Preserving Projection (B2DDLPP)

Bilinear two-dimensional discriminant locality preserving projection (B2DDLPP) method is based on the matrix-variate Gaussian model. This model denotes that within-class covariance between any two spatial-spectral features can be decomposed into two parts.

Exchanging the numerator and denominator in the objective function of the 2DDLPP algorithm, the objective function of B2DDLPP can be reformed as follows:

(15) $$\begin{array}{c} J\left(G\right)=\frac{G^{T}FHF^{T}G}{G^{T}XLX^{T}G}=\frac{G^{T}S_{b}G}{G^{T}S_{w}G}, \end{array}$$

where

(16) $$\begin{array}{c} S_{w}=\frac{1}{2}\sum_{s=1}^{Z}\sum_{i,j=1}^{n_{s}}\left(X_{i}^{s}-X_{j}^{s}\right){\left(X_{i}^{s}-X_{j}^{s}\right)}^{T}W_{ij}^{s},\\ S_{b}=\frac{1}{2}\sum_{i,j=1}^{Z}\left(F_{i}-F_{j}\right){\left(F_{i}-F_{j}\right)}^{T}B_{ij}. \end{array}$$

The projection directions can be obtained by maximizing the objective function, satisfying

(17) $$\begin{array}{c} G=\arg\underset{G}{\max}\frac{G^{T}FHF^{T}G}{G^{T}XLX^{T}G}. \end{array}$$

Combining moment estimation of separable covariance matrix in separable LDA [29] and the high similarity between LDA and DLPP [30], we use the two following equations to estimate the corresponding within-class covariance matrices. Decompose **S**_*w*_ to get

(18) $$\begin{array}{c} \psi =\frac{1}{2*N_{f}}\sum_{s=1}^{Z}\sum_{i,j=1}^{n_{s}}{\left(X_{i}^{s}-X_{j}^{s}\right)}^{T}\left(X_{i}^{s}-X_{j}^{s}\right)W_{ij}^{s}, \end{array}$$

(19) $$\begin{array}{c} \phi =\frac{1}{2*N_{g}}\sum_{s=1}^{Z}\sum_{i,j=1}^{n_{s}}\left(X_{i}^{s}-X_{j}^{s}\right){\left(X_{i}^{s}-X_{j}^{s}\right)}^{T}W_{ij}^{s}, \end{array}$$

where **X**_*i*_^*s*^ denotes the *i*th feature matrix in class *s*, **X**_*j*_^*s*^ denotes the *j*th feature matrix in class *s*, **W**_*ij*_^*s*^ is the weight between **X**_*i*_^*s*^ and **X**_*j*_^*s*^, and it is defined as **W**_*ij*_^*s*^=exp(−‖**X**_*i*_^*s*^ − **X**_*j*_^*s*^‖^2^/*t*). *n*_*s*_ is the number of samples in the *s*th class. *Z* is the total number of classes. T is the transpose operation of the matrix.

Besides, the between-class scatter matrix can be considered as a separable structure. Vectorize the mean matrix in each class to get SB= SBR⊗SBL, where

(20) $$\begin{array}{c} S_{B}=\frac{1}{2}\sum_{a,b=1}^{Z}\left(u_{a}-u_{b}\right){\left(u_{a}-u_{b}\right)}^{T}B_{ab}, \end{array}$$

(21) $$\begin{array}{c} S_{BL}=\frac{1}{2}\sum_{a,b=1}^{Z}\left(F_{a}-F_{b}\right){\left(F_{a}-F_{b}\right)}^{T}B_{ab}, \end{array}$$

(22) $$\begin{array}{c} S_{BR}={\text{tr}}^{-1}\left(S_{BL}\right)*\frac{1}{2}\sum_{a,b=1}^{Z}{\left(F_{a}-F_{b}\right)}^{T}\left(F_{a}-F_{b}\right)B_{ab}, \end{array}$$

where **u** denotes the vectorization of **F**; *u* = vecF. **F**_*s*_ is the mean matrix of the *s*th class: **F**_*s*_=(1/*n*_*s*_)∑_*i*=1_^*n*_*s*_^**X**_*i*_^*s*^. **B** is the weight matrix between any two classes' mean matrix and it is defined as

(23) $$\begin{array}{c} B_{ab}=\exp\left(\frac{-{\left\|F_{a}-F_{a}\right\|}^{2}}{t}\right). \end{array}$$

Using this assumption, we obtain the eigenvalues and eigenvectors of *ϕ*-1SBL, denoted by *λ*l and ul, respectively. Similarly, we obtain the eigenvalues and the eigenvectors of Ψ-1SBR, denoted by *γ*j and vj. Then, sort the two eigenvalues *λ*l and *γ*j in a descending order. The corresponding projection matrix can be constructed, respectively: **U**=[**u**_1_, **u**_2_,…, **u**_*N*_*f*__] and **V**=[**v**_1_, **v**_2_,…, **v**_*N*_*g*__]. Thus, the feature matrix Y is defined as

(24) $$\begin{array}{c} Y=U^{T}XV. \end{array}$$

Finally, in order to get the d-dimensional features, we choose the yij elements of Y which correspond to the *d* largest *λ*_*l*_*γ*_*j*_ values.

### 2.3. Materials and Experiments

#### 2.3.1. Preprocessing and the Flow Chart of the Experiment

**Figure 1:**
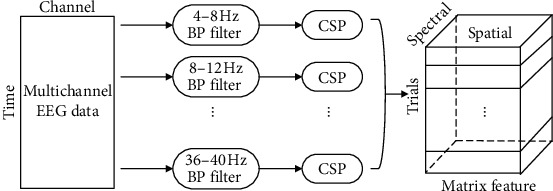
Operation steps of FBCSP. Each trial in matrix feature contains spatial-spectral information.

We conducted feature extraction using the proposed B2DDLPP in three databases. B2DDLPP algorithm is compared with LDA, two-dimensional linear discriminant analysis (2DLDA), DLPP, and 2DDLPP. It should be noted that the same preprocessing method was applied to three data sets in this experiment: Before using the three feature extraction methods mentioned above, we apply the one-versus-rest (OVR) multiclass extension of the filter bank common spatial patterns (FBCSP) [31, 32] method to process the data. Taking four classes as an example, we take one class of samples as positive samples and the remaining samples as negative samples and perform FBCSP operations on the data to obtain a set of features. By analogy, a total of four sets of features can be obtained. Combine the four groups of features, and finally get a feature matrix of*N*_*f*_ × *N*_*g*_size, where *N*_*f*_ is the number of frequency bands in FBCSP (9 in this article), *N*_*g*_= 2 × *m* × Numclass, *m* is the number of pairs of features, and Numclass is the total number of classes. This method can reduce the dimensionality of the EEG data beforehand to ensure that the feature dimension of the data is less than the number of samples. This allows the subsequent feature extraction method to proceed smoothly. At the same time, effective spatial structure and frequency information are retained to obtain better classification results. As alpha band (8–13 Hz) and beta band (14–30 Hz) contain rich information for MI task, we divide all EEG signals into frequency subbands. The FBCSP employs a filter bank that covers 4–40 Hz, which comprises 9 bandpass filters that cover 4 Hz each [31]; and, in order to get a flatter delay response and low signal distortion, we use 6-order Chebyshev type II filter in this paper. The specific process is shown in Figure 1.

Considering that SVM classifier has been widely used in EEG classification [33–35], we use SVM classifier for classification in this paper. We divide the data set into two sets: training set and test set. The performance of BCI algorithms highly depends on the dimensionality of the feature space at the classifier's input, denoted by *d*. For each method, the optimal dimensionality *d*_op_ of the feature space in training set is determined based on the average performance of each subject over all the validation runs. The feature space dimensionality for each method in test set is set based on the value of *d*_op_ in the validation phase. In order to make different methods more suitable for the data, so as to get the optimal dimension *d*_op_, the selection range of parameter *m* in the preprocessing method is from 1 to 4. The value of the parameter *m* affects the data dimension after preprocessing.

**Figure 2:**
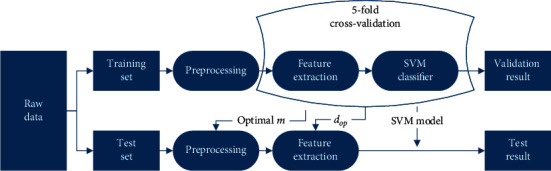
The flow chart of the experiment.

The flow chart of the experiment is shown in Figure 2. All experiments are performed on MATLAB R2017a and Windows 10, with AMD core 2600X CPU and 16 GB RAM.

#### 2.3.2. Data Set

*(1) Set 1: BCI competition IV, data sets 2a (Exp.1)*. The ultimate purpose of this experiment is to classify the following motor-imagery tasks: left hand, right hand, feet, and tongue movement. This data set contains EEG signals of nine healthy subjects. It is recorded in two sessions and the signals are recorded using 22 Ag/AgCl electrodes at 250 Hz sampling rate. Each session consists of six runs and each of which includes 48 trials of a length of 3 seconds, yielding a total of 288 trials per session. These two sessions are used as training set and test set, respectively. It should be mentioned that there are three Electrooculogram (EOG) channel recordings in this data set and they can be used as a reference for denoising. In this paper, we chose the time period of 3 s to 6 s and in order to preserve complete spatial information, all channels are reserved.

*(2) Set 2: BCI competition III, data sets IIIa (Exp.2)*. This data set consists of recordings from three healthy subjects (k3b, k6b, and l1b). Each subject sat in a relaxing chair with armrests and was asked to perform imagery movements with four different tasks: left hand, right hand, foot, and tongue. Each subject completed 60 trials per class. Recordings were made with a 60-channel EEG amplifier from Neuroscan with the left mastoid for reference and the right mastoid as ground. EEG signals were recorded with a sampling rate of 250 Hz and filtered between 1 and 50 Hz with the notch filter on. In this paper, we chose the time period of 3 s to 7 s and all channels are reserved.

*(3) Set 3: data sets 3 (Exp.3)*. The third data set used in this paper was obtained by our laboratory. This data set consists of recordings from 10 subjects and each subject sat in a relaxing chair with armrests and was asked to perform imagery movements with three different tasks: left hand, right hand, and the idle state. Motor imaging duration is 4 seconds. The signals are recorded using 62 Ag/AgCl electrodes at 1000 Hz sampling rate and each subject completed 125 trials per class, yielding a total of 375 trials. Among them, 300 trials are used as the training set, and the remaining data are used as the test set. In order to reduce data redundancy and improve the efficiency of data processing, we performed downsampling to reduce the frequency to 250 Hz.

## 3. Results and Discussion

In this section, we show the results of different methods on training set and the test set. We used a 5-fold cross-validation method to process the training set and then use the optimal parameters obtained in the training set for the classification of the test set. All results are displayed by the classification accuracy.

The pseudocode for training the B2DDLPP feature extractor is presented in Table 1 and the validation results of training set for Exp.1, Exp.2, and Exp.3 are presented in Tables 2–4, respectively. For each subject, the highest average recognition rate over all the cross-validation runs and the corresponding feature dimension of different algorithms are reported. We also studied the case where no feature extraction method is utilized and the FBCSP features are directly passed to the classifier.

Table 2 shows that the average recognition accuracy of B2DDLPP is improved by 2.81%, 8.47%, 7.07%, 4.86%, and 3.8% compared to those of None, LDA, 2DLDA, DLPP, and 2DDLPP, respectively. From the results, B2DDLPP has the highest accuracy rate for each subject; and whether to use 2D method or manifold learning method, it can be seen from the comparison result that these two factors have an important influence on the accuracy of EEG classification. In the operation of LDA and DLPP, vectorization of the extracted matrix features will cause the loss of important information. There is an indispensable connection in the matrix structure, which has a great influence on the accuracy of classification. The accuracy of 2DLDA and 2DDLPP using the 2D method has improved compared with the method without 2D processing; and, compared to 2DLDA, the accuracy of 2DDLPP increased by 3.27%, which shows that keeping locality-geometric structure of EEG data in a neighboring area plays an effective role in improving the classification accuracy.

When compared with 2DDLPP, the result of B2DDLPP has greatly improved. 2DDLPP only transforms the rows or columns of matrix data, which inevitably leads to unnecessary information loss. B2DDLPP uses bilinear structure to decompose the covariance matrix into row part and column part, which has a better grasp of the internal interconnections of spatial data. Therefore, B2DDLPP can extract more discriminative information. We can draw the same conclusion in Table 3.

The overall recognition accuracy in Table 4 is not as high as that in the other two tables. The reason may be that the subject of this data set is not a professionally trained person having a long-term specialized motor imaging training. This insufficient effective information in the original data leads to the result, whereas, in Exp.1 and Exp.2, the subject is the specially trained group of people, which generates less noise when performing a motor imaging task. Table 4 shows that the average recognition accuracy of B2DDLPP is improved by 2.34%, 6.75%, 5.7%, 4.66%, and 3.66% compared to those of None, LDA, 2DLDA, DLPP, and 2DDLPP, respectively. This result also fully proves the effectiveness of the 2D method and manifold learning method for the extraction of spatial and structural information from EEG signals.

It can be seen from the results of the three tables that although B2DDLPP has the highest accuracy rate, the classification accuracy of other feature extraction methods listed in the table is a bit lower than the accuracy rate without feature extraction. The reason can be attributed to the two following points: First, the methods of LDA and DLPP are not very robust to noise. Among the acquisition channels, the channels related to motor imagery are only a part of them. In this experiment, in order to ensure the integrity of the channel structure, we use the data of all channels, which brings redundant data and noise. Second, the extraction of effective information is insufficient. 2DLDA and 2DDLPP methods only transform the rows or columns of matrix data and this one-sided compression is not sufficient for the extraction of effective features in the spatial-spectral matrix.

It should be noted that the parameter *t* related to the calculation of the weight matrix in DLPP, 2DDLPP, and B2DDLPP is set to 1.

**Figure 3:**
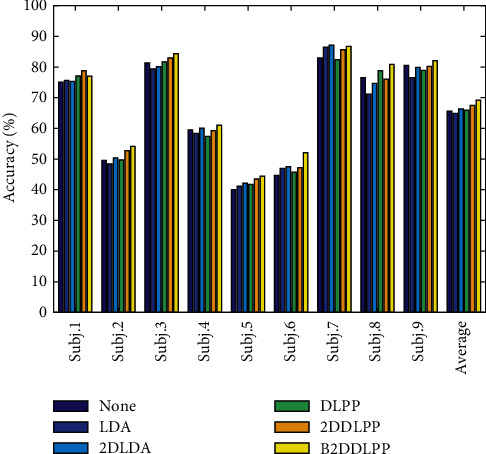
The results of the test set for Exp.1. The figure shows the accuracy of 9 subjects and the average accuracy of them.

**Figure 4:**
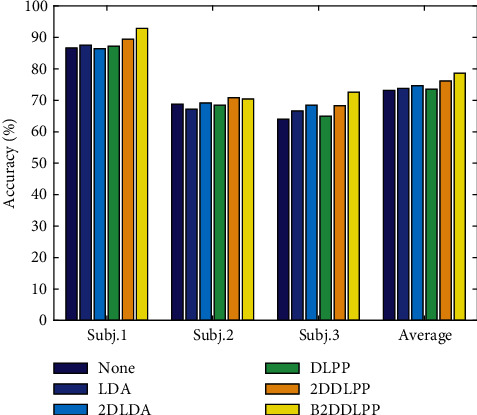
The results of the test set for Exp.2.

The following shows the results of the test set. The results of test set for Exp.1 and Exp.2 are presented in Figures 3 and 4, respectively. Note that the feature space dimensionality for each method in test set is set based on the value of *d*_op_ in the validation phase.

We can see from the figures that the performance results on the test data show a trend that is very similar to that of the performance results during the cross-validation phase.

**Figure 5:**
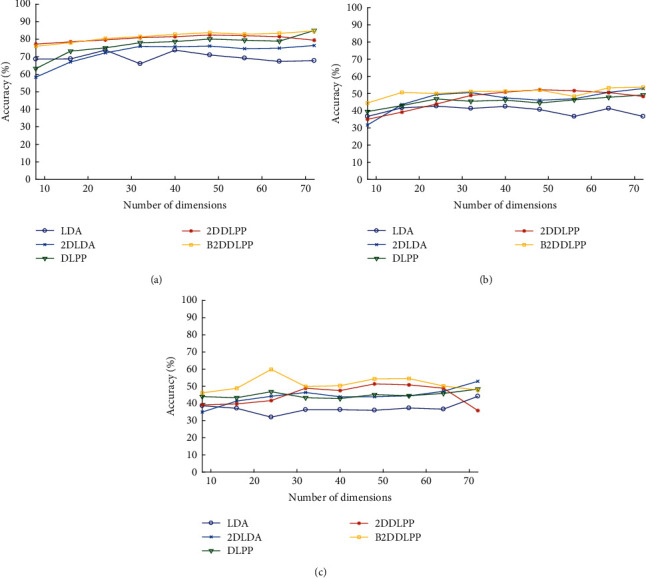
Five-fold cross-validation performance on different number of dimensions on Exp.2: (a) the accuracy of different dimensions in subj.1; (b) the accuracy of different dimensions in subj.2; (c) the accuracy of different dimensions in subj.3.

In addition, we studied the influence of different feature dimensions on the effects of these methods.  Figure 5 shows recognition accuracy of all the methods on different number of dimensions under three subjects in Exp.2. The results in the figure are shown by the average accuracy of 5-fold cross-validation. We can see from the figure that the overall trend of various methods rises slightly with the increase of the dimension and finally tends to be flat. Combining the curve results of the three subjects, the accuracy of the B2DDLPP method is the highest, followed by 2DDLPP. The results of DLPP and 2DLDA are not much different, and the worst is the LDA method. Different methods have different extraction capabilities for spatial information and structural information and the effect of this extraction determines the final accuracy. The results also show that B2DDLPP can achieve a high accuracy rate in the case of low feature dimensions, which can greatly help reduce the complexity of data and extract effective features.

## 4. Conclusions

In this paper, we propose a B2DDLPP algorithm. By adding the matrix-variate Gaussian model and a bilinear structure, B2DDLPP decomposes the within-class covariance matrix and the between-class scatter matrix to obtain the optimal projection matrix. As a matrix-based method, B2DDLPP is more effective for extracting spatial information than vector-based methods. At the same time, the bilinear structure further enhances this effect.

In order to fully verify the effectiveness of the algorithm, we apply the B2DDLPP algorithm to three EEG data sets in this paper. The results show that B2DDLPP has a higher feature extraction performance compared to other methods.

## Figures and Tables

**Table 1:** The pseudocode for training the B2DDLPP feature extractor.

Algorithm: B2DDLPP
Inputs:
- Training sample *X*_*N*_*f*_×*N*_*g*__. The total number of samples is *N*. The number of training samples in each class is *N*_*i*_, 1≤ *i* ≤ *Z*.
Outputs:
- The feature extraction operators *U*_*N*_*f*_×*N*_*f*__and *V*_*N*_*g*_×*N*_*g*__- the corresponding *λ*_*l*_ and *γ*_*j*_ values which determine the priority in selecting the elements in feature matrix.
Procedure:
1. Calculate the spatial covariance matrix *ψ* and the spectral covariance matrix *ϕ* according to (18) and (19).
2. Calculate *S*_*BL*_ and *S*_*BR*_ according to (21) and (22).
3. Calculate the eigenvalues *λ*_*l*_ and the corresponding eigenvectors *u*_*l*_ of *ϕ*^−1^*S*_*BL*_, 1≤ *l* ≤*N*_*f*_. And calculate the eigenvalues *γ*_*j*_ and the corresponding eigenvectors *v*_*j*_ of *ψ*^−1^*S*_*BR*_4. Construct U and V.
5. Calculate the feature matrix Y according to (24).
6. Choose the *y*_*lj*_ elements of Y which correspond to the *d* largest *λ*_*l*_*γ*_*j*_ values. D is the dimension after feature extraction.

**Table 2:** Cross-validation performance results for different algorithms in Exp.1.

Feature extraction	Subj.1 (%)	Subj.2 (%)	Subj.3 (%)	Subj.4 (%)	Subj.5 (%)	Subj.6 (%)	Subj.7 (%)	Subj.8 (%)	Subj.9 (%)	Average (%)
None	84.72 m = 3	57.70 m = 1	87.23 m = 2	54.93 m = 4	63.81 m = 2	50.71 m = 4	88.61 m = 1	**87.84** m = 4	81.05 m = 1	72.88
LDA	79.22 m = 1	51.78 m = 1	79.57 m = 1	50.38 m = 1	63.94 m = 1	44.81 m = 1	85.91 m = 1	75.04 m = 1	74.34 m = 1	67.22
	*d*_op_=72	*d*_op_=64	*d*_op_=53	*d*_op_=47	*d*_op_=7	*d*_op_=6	*d*_op_=10	*d*_op_=22	*d*_op_=58	
2DLDA	83.27 m = 1	55.19 m = 2	78.44 m = 2	50.33 m = 1	67.32 m = 2	44.56 m = 1	86.10 m = 1	77.43 m = 2	74.97 m = 1	68.62
	*d*_op_=6	*d*_op_=5	*d*_op_=4	*d*_op_=4	*d*_op_=3	*d*_op_=5	*d*_op_=4	*d*_op_=7	*d*_op_=5	
DLPP	80.55 m = 2	55.56 m = 1	84.38 m = 1	54.17 m = 2	63.20 m = 1	50.69 m = 2	88.19 m = 1	80.55 m = 2	80.21 m = 2	70.83
	*d*_op_=134	*d*_op_=65	*d*_op_=72	*d*_op_=106	*d*_op_=69	*d*_op_=122	*d*_op_=51	*d*_op_=132	*d*_op_=128	
2DDLPP	84.03 m = 1	56.40 m = 1	86.60 m = 1	52.71 m = 3	65.89 m = 2	49.66 m = 2	88.97 m = 1	81.79 m = 2	80.98 m = 1	71.89
	*d*_op_=6	*d*_op_=7	*d*_op_=7	*d*_op_=9	*d*_op_=9	*d*_op_=6	*d*_op_=9	*d*_op_=7	*d*_op_=9	
B2DDLPP	**86.80** m = 2	**61.11** m = 1	**89.61** m = 1	**58.64** m = 2	**71.91** m = 2	**53.48** m = 1	**90.61** m = 1	85.38 m = 4	**83.68** m = 1	**75.69**
	*d*_op_=142	*d*_op_=58	*d*_op_=44	*d*_op_=126	*d*_op_=70	*d*_op_=67	*d*_op_=63	*d*_op_=167	*d*_op_=52	

For each method and each subject, optimal *m* related to FBCSP's output and the optimal dimension (*d*_op_) are presented.

**Table 3:** Cross-validation performance results for different algorithms in Exp.2.

Feature Extraction	Subj.1 (% m, *d*_op_)	Subj.2 (% m, *d*_op_)	Subj.3 (% m, *d*_op_)	Average (%)
None	81.67 m = 1	56.67 m = 1	58.33 m = 2	65.56
LDA	78.89 m = 1,*d*_op_=59	58.33 m = 2,*d*_op_=37	53.33 m = 2,*d*_op_=103	63.52
2DLDA	79.89 m = 2,*d*_op_=8	58.50 m = 2,*d*_op_=3	55.17 m = 2,*d*_op_=3	64.52
DLPP	85.00 m = 1,*d*_op_=58	57.50 m = 1,*d*_op_=22	52.50 m = 1,*d*_op_=30	65.00
2DDLPP	85.44 m = 3,*d*_op_=5	54.33 m = 1,*d*_op_=7	57.67 m = 1,*d*_op_=7	65.81
B2DDLPP	**87.22** m = 1,*d*_op_=37	**60.00** m = 2,*d*_op_=79	**64.17** m = 1,*d*_op_=26	**70.46**

For each method and each subject, optimal m related to FBCSP's output and the optimal dimension (*d*_op_) are presented.

**Table 4:** Cross-validation performance results for different algorithms in Exp.3.

Feature extraction	Subj.1 (%)	Subj.2 (%)	Subj.3 (%)	Subj.4 (%)	Subj.5 (%)	Subj.6 (%)	Subj.7 (%)	Subj.8 (%)	Subj.9 (%)	Subj.10 (%)	Average (%)
None	43.33 m = 1	79.67 m = 1	58.31 m = 2	50.66 m = 2	48.33 m = 2	41.00 m = 2	57.60 m = 2	42.36 m = 1	39.33 m = 1	84 m = 1	54.45
LDA	39.33 m = 1	73.67 m = 1	50.00 m = 1	45.00 m = 1	43.34 m = 1	40.33 m = 1	54.77 m = 1	39.67 m = 1	39.00 m = 1	75.33 m = 1	50.04
	*d*_op_=23	*d*_op_=18	*d*_op_=39	*d*_op_=49	*d*_op_=28	*d*_op_=41	*d*_op_=11	*d*_op_=50	*d*_op_=52	*d*_op_=24	
2DLDA	41.13 m = 1	72.33 m = 1	52.67 m = 1	47.52 m = 1	45.67 m = 1	39.83 m = 1	54.93 m = 1	40.33 m = 1	39.13 m = 1	77.33 m = 1	51.09
	*d*_op_=5	*d*_op_=3	*d*_op_=4	*d*_op_=6	*d*_op_=2	*d*_op_=2	*d*_op_=1	*d*_op_=4	*d*_op_=2	*d*_op_=5	
DLPP	41.67 m = 2	75.33 m = 1	52.67 m = 2	46.00 m = 2	49.33 m = 2	39.33 m = 1	56.00 m = 2	39.67 m = 2	39.67 m = 1	81.67 m = 1	52.13
	*d*_op_=98	*d*_op_=11	*d*_op_=44	*d*_op_=8	*d*_op_=20	*d*_op_=29	*d*_op_=23	*d*_op_=98	*d*_op_=20	*d*_op_=7	
2DDLPP	41.67 m = 1	78.00 m = 1	55.33 m = 2	47.33 m = 2	45.33 m = 1	40.67 m = 1	55.33 m = 2	41.67 m = 2	40.33 m = 2	79.67 m = 1	53.13
	*d*_op_=2	*d*_op_=2	*d*_op_=7	*d*_op_=9	*d*_op_=2	*d*_op_=7	*d*_op_=7	*d*_op_=9	*d*_op_=5	*d*_op_=3	
B2DDLPP	**45.67** m = 1	**80.67** m = 2	**59.33** m = 2	**51.67** m = 2	**52.82** m = 2	**45.00** m = 2	**59.70** m = 2	**44.40** m = 2	**42.33** m = 1	**86.33** m = 1	**56.79**
	*d*_op_=35	*d*_op_=82	*d*_op_=51	*d*_op_=45	*d*_op_=94	*d*_op_=14	*d*_op_=19	*d*_op_=79	*d*_op_=49	*d*_op_=54	

For each method and each subject, optimal *m* related to FBCSP's output and the optimal dimension (*d*_op_) are presented.

## Data Availability

The Exp1 and Exp2 data used to support the findings of this study are available at http://www.bbci.de/competition/.
